# Decreasing hill density combined with increasing nitrogen rate led to yield decline in hybrid rice under low-light conditions

**DOI:** 10.1038/s41598-019-52376-2

**Published:** 2019-10-31

**Authors:** Hejun Ao, Xiaobing Xie, Min Huang, Yingbin Zou

**Affiliations:** 1 Crop and Environment Research Center, College of Agronomy, Hunan Agricultural University, Changsha, 410128 China; 2 Key Laboratory of Crop Physiology, Ecology and Genetic Breeding of Ministry of Education, Jiangxi Agricultural University, Nanchang, 330045 China

**Keywords:** Light responses, Agroecology

## Abstract

Low light is a common environmental factor that adversely affects rice yields. This study was conducted to evaluate the combined effect of hill density and nitrogen (N) fertilizer rate on yield attributes in hybrid rice under low-light conditions. Field experiments were conducted in 2014 and 2015. Two hybrid rice cultivars (Y-liangyou 1 and Luoyou 9348) were grown under combinations of three hill density levels (high, 40 × 10^4^ hills ha^−1^; moderate, 27 × 10^4^ hills ha^−1^; low, 14 × 10^4^ hills ha^−1^) and two N rate levels (high, 240 kg ha^−1^; moderate, 143–148 kg ha^−1^), and shaded from heading to maturity. Grain yield was highest in the combination of high hill density and moderate N rate and significantly declined with decreasing hill density combined with increasing N rate for both cultivars in both years. Averaged across two cultivars and two years, grain yield declined by about 4% for each 10% decrease in hill density combined with each 10% increase in N rate. A significant reduction in spikelet filling percentage was observed with decreasing hill density combined with increasing N rate in Y-liangyou 1 in 2015 and Luoyou 9348 in 2014. The same trend was observed for grain weight in Y-liangyou 1 in 2014 and Luoyou 9348 in 2015. These results indicate that adopting the practice of decreasing hill density combined with increasing N rate can result in poor grain filling and consequently yield decline in hybrid rice under low-light conditions.

## Introduction

Rice is the staple food crop for more than 65% of the population in China^1^. To ensure national food security, great efforts have been made toward rice improvement in China over the past several decades, accomplishing breakthroughs including the development of semi-dwarf rice cultivars in the late 1950s and hybrid rice cultivars in the late 1970 s^2^. However, rice yield is dependent not only on cultivars but also on the environment in which it is cultivated^3,4^.

Light is one of most important environmental variables determining rice yields. Low light can negatively affect several physiological processes (*e.g*., photosynthesis) and hence cause yield loss in rice^5^. Consecutive days of cloudy and/or rainy weather is a common low-light event in rice production. In August 2014, a phase of cloudy and rainy weather lasted for 15 days in the middle and lower reaches of the Yangtze River of China^6^, during grain filling for the single-season rice in the region. Additionally, as a result of intensive air pollution, China experiences severe haze pollution, which can result in low light intensity with negative effects on rice yields^7,8^. It is estimated that the regional haze pollution leads to about a 2% reduction in total rice production in China^7^. These facts highlight the need for optimizing crop management to minimize the potential negative yield effects of low light on rice.

Plant density and nitrogen (N) rate are two key factors in crop management. These two factors can directly affect the plant population size and may further alter the effects of low light on rice. There have been reports on the single effects of plant density or N rate on yield attributes in rice under low-light conditions^9,10^, but limited information is available on their combined effects. Such information is useful because plant density and N rate are always adjusted simultaneously in rice production. For example, many Chinese rice farmers prefer to adopt the practice of decreasing hill density and increasing N rate to save labor, the availability of which has declined markedly in China due to the urban expansion^2^. On the contrary, several studies recommend adopting the combined practice of increasing plant density and decreasing N rate for rice production^11,12^ because a high N rate has diminishing returns with sharply decreased N use efficiency^13^, as well as imposing substantial environmental costs such as enhanced N deposition^14^, soil acidification^15^, and surface water eutrophication^16^.

In this study, field shading experiments were conducted in two years to determine grain yield and yield components in two hybrid rice cultivars grown under different hill densities and N rates. The objective of this study was to evaluate the combined effect of hill density and N rate on yield attributes in hybrid rice under low-light conditions.

## Results

**Figure 1:**
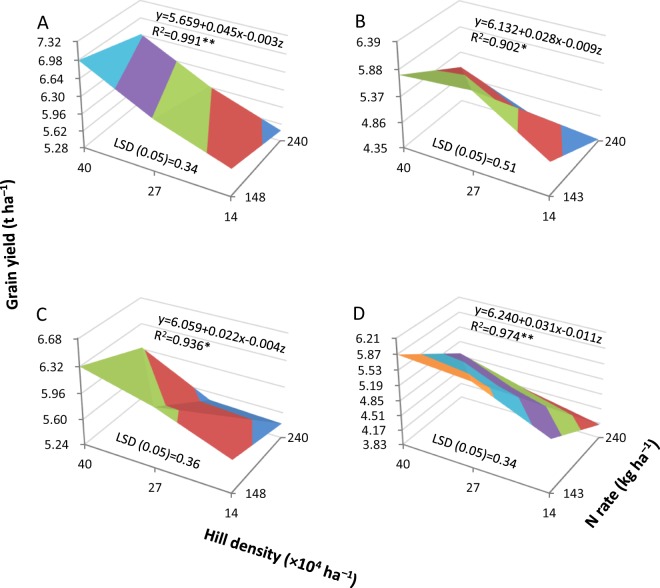
Grain yield in two hybrid rice cultivars, Y-liangyou 1 (**A**,**B**) and Luoyou 9348 (**C**,**D**), as affected by combinations of hill density and N rate under low-light conditions in 2014 (**A**,**C**) and 2015 (**B**,**D**). Data describing grain yield (ordinate) are separated by the LSD (0.05) value (the least significant difference at the 0.05 probability level) and color coded. * and ** denote significant relationships between grain yield (y) with hill density (x) and N rate (z) at the 0.05 and 0.01 probability levels, respectively.

Grain yield was significantly affected by hill density and N rate for both Y-liangyou 1 and Luoyou 9348 in both 2014 and 2015 (Fig. 1A–D). Grain yield was highest in the combination of high hill density (40 × 10^4^ hills ha^−1^) and moderate N rate (143–148 kg ha^−1^) and declined with decreasing hill density combined with increasing N rate for both cultivars in both years. More than 90% of the yield variation was explained by the combined effect of hill density and N rate. Averaged across cultivars and years, grain yield declined by about 4% for each 10% decrease in hill density combined with each 10% increase in N rate.

**Figure 2:**
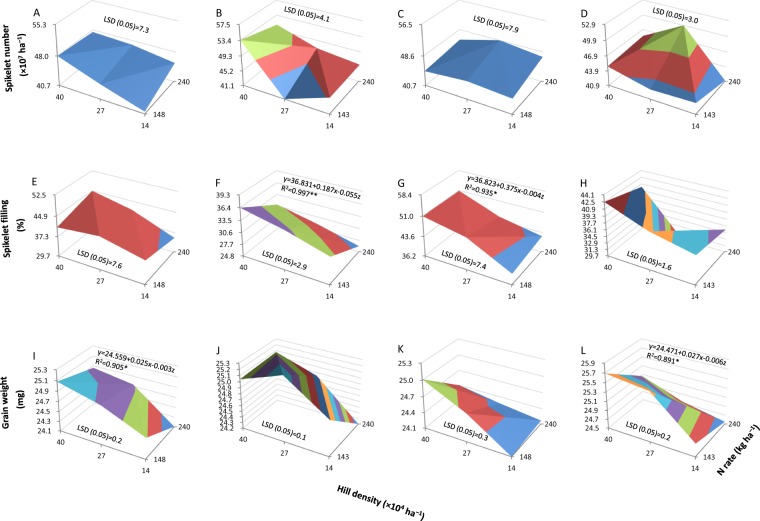
Spikelet number (**A**–**D**), spikelet filling percentage (**E**–**H**), and grain weight (**I**–**L**) in two hybrid rice cultivars, Y-liangyou 1 (**A**,**B**,**E**,**F**,**I**,**J**) and Luoyou 9348 (**C**,**D**,**G**,**H**,**K**,**L**), as affected by combinations of hill density and N rate under low-light conditions in 2014 (**A**,**C**,**E**,**G**,**I**,**K**) and 2015 (**B**,**D**,**F**,**H**,**J**,**L**). Data describing yield attributes (ordinate) are separated by the LSD (0.05) value (the least significant difference at the 0.05 probability level) and color coded. * and ** denote significant relationships between yield attributes (y) with hill density (x) and N rate (z) at the 0.05 and 0.01 probability levels, respectively.

The effect of hill density and N rate on spikelet number in both Y-liangyou 1 and Luoyou 9348 was insignificant in 2014 but significant in 2015 (Fig. 2A–D). In 2015, the highest spikelet number was observed in the combination of high hill density (40 × 10^4^ hills ha^−1^) and moderate N rate (143 kg ha^−1^) for Y-liangyou 1 and in the combination of moderate hill density (27 × 10^4^ hills ha^−1^) and high N rate (240 kg ha^−1^) for Luoyou 9348 (Fig. 2B,D).

Spikelet filling percentage and grain weight were significantly affected by hill density and N rate for both Y-liangyou 1 and Luoyou 9348 in both 2014 and 2015 (Fig. 2E–L). In general, spikelet filling percentage and grain weight tended to decrease with decreasing hill density combined with an increasing N rate. This trend was significant for spikelet filling percentage in Y-liangyou 1 in 2015 and Luoyou 9348 in 2014 (Fig. 2F,G), and for grain weight in Y-liangyou 1 in 2014 and Luoyou 9348 in 2015 (Fig. 2I,L).

## Discussion

The present study showed that decreasing hill density combined with increasing N rate caused a yield decline in hybrid rice grown under shading during the ripening period. This finding is not in agreement with patterns observed under favorable conditions^11,12^, in which there is a yield compensation effect between hill density and N rate, *i.e*., rice crops grown under the combination of low hill density and high N rate produced a similar grain yield to the combination of high hill density and low N rate.

The above contradiction indicates that adopting the practice of increasing hill density combined with decreasing N rate in rice production has the potential benefit of avoiding large variation in grain yield across diverse environments, *i.e*., high yield stability. This is a key goal of agricultural progress and has become even more important as our climate is changing and the intensity and frequency of extreme weather events may increase in the future^17^. Moreover, this practice has been recommended by several studies^11,12^ because the use of large amounts of N in crop production in China has led to diminishing returns^13^ and substantial environmental costs^14–16^. However, many Chinese farmers do not prefer this practice under manual work conditions because the operation of dense transplanting requires a large amount of manpower, which is a limited resource in China due to the urban expansion^2^. Further, the task of dense transplanting is very laborious, involving working in a stooping posture and moving through a muddy field^18^. This highlights the need for developing labor-saving methods of dense transplanting, *e.g*., mechanically dense transplanting, for rice production in China.

Decreasing hill density combined with increasing N rate led to decreases in spikelet filling percentage or grain weight in hybrid rice in this study. The decreased spikelet filling percentage was mainly due to an increase in the number of partially filled spikelets, which were categorized as unfilled spikelets in the water separation analysis (personal observation). These observations indicate that poor grain filling was responsible for the yield decline caused by decreasing hill density combined with increasing N rate in hybrid rice grown under shading during the ripening period. It has been well-documented that grain filling is closely linked to the whole-plant senescence process in rice, and unfavorably delayed senescence can result in large amounts of non-structural carbohydrate (NSC) left in the straw, and lead to a low grain-filling rate and consequently many poorly filled grains^19,20^. High N rate is one of the common factors causing unfavorably delayed senescence in rice production^19,20^. In addition, it has been reported that rice grain filling is also affected by hill density, *i.e*., decreasing hill density can lead to decreased spikelet filling percentage and grain weight by decreasing NSC in the leaf sheath plus the stem at the full heading stage^21^. These mechanisms may also explain the poor grain filling caused by decreasing hill density combined with increasing N rate in hybrid rice in the present study. The results of this study highlight the need for a greater fundamental understanding of the combined effect of hill density and N rate on physiological processes governing grain filling in hybrid rice grown under low-light conditions.

Spikelet number is considered the primary determinant of grain yield in rice under favorable conditions^22^, and plant density and N rate are two key crop management factors for achieving a high spikelet number^12,21,23^. In the present study, although the significance of the combined effect of hill density and N rate on spikelet number varied across years for both tested cultivars, the trend was consistent, *i.e*., the highest spikelet number was achieved in the combination of high hill density and moderate N rate for Y-liangyou 1 and in the combination of moderate hill density and high N rate for Luoyou 9348 (Fig. 2A–D). The cultivar difference in response to combinations of hill density and N rate may be related to plant type and to N sensitivity. Y-liangyou 1 is a cultivar with a compact plant type (erect leaves) and lower N sensitivity, while Luoyou 9348 is a cultivar with a loose plant type (droopy leaves) and higher N sensitivity. These facts suggest that the regulating effect of hill density and N rate on spikelet number is dependent on cultivar characteristics.

## Methods

Field experiments were conducted in Yongan (28°09′N, 113°37′E, 43 m asl), Hunan Province, China in 2014 and 2015. This site is located in the middle reach of the Yangtze River and has a moist subtropical monsoon climate. Average daily maximum and minimum temperatures and incident solar radiation during the rice-growing season were 31.0 °C, 24.4 °C and 13.6 MJ m^−2^ in 2014 and 30.3 °C, 23.1 °C and 16.1 MJ m^−2^ in 2015, respectively. The soil of the experimental field has a clay texture with the following soil properties at the 0–20 cm layer: pH 6.22, 44.8 g organic matter kg^−1^, 1.24 g total N kg^−1^, 0.70 g total phosphorus (P) kg^−1^, 5.68 g total potassium (K) kg^−1^, 147 mg available N kg^−1^, 25.2 mg available P kg^−1^, and 123 mg available K kg^−1^.

Two hybrid rice cultivars, Y-liangyou 1 and Luoyou 9348, were grown under six combinations of hill density and N rate each year. Y-liangyou 1 is a two-line *indica* hybrid cultivar (Y58S × R9311) developed by the Hunan Hybrid Rice Research Center, China. Luoyou 9348 is a three-line *indica* hybrid cultivar (Luohong 4 A × Chenghui 9348) developed by the Wuhan Guoying Seed Co., Ltd., and the Wuhan University, China. These two cultivars were selected because they have been widely grown in the middle and lower reaches of the Yangtze River. The hill density was set at three levels: high (40 × 10^4^ hills ha^−1^), moderate (27 × 10^4^ hills ha^−1^), and low (14 × 10^4^ hills ha^−1^). The N rate was set at two levels: high (240 kg ha^−1^ in both 2014 and 2015) and moderate (148 kg ha^−1^ in 2014 and 143 kg ha^−1^ in 2015). The different N rates used for the moderate level fertilization between years were adjusted according to the chlorophyll meter (SPAD-502, Minolta, Osaka, Japan) readings on the upmost fully expanded leaves^4^, which were 34.4–39.9 in 2014 and 36.6–41.4 in 2015, and which determined the N rate of panicle fertilizer. Low-light treatment was performed from heading to maturity by covering plots with sunshade nets at a height of 2.5 m above the ground, which caused a reduction of approximately 70% in the canopy light. The daily incident solar radiation during the period from heading to maturity was 12.6 and 15.4 MJ m^−2^ in 2014 and in 2015, respectively. The experiment was arranged in a split plot design with cultivars as the main plots and combinations of hill density and N rate as subplots, with three replicates and a subplot size of 10 m^2^.

Pre-germinated seeds were sown in a seedbed on 10 and 13 May in 2014 and 2015, respectively. Transplanting was done with two seedlings per hill for all three hill densities on 5 and 7 June in 2014 and 2015, respectively. N was split-applied as basal (60–120 kg ha^−1^), tillering (45–48 kg ha^−1^), and panicle fertilizer (38–72 kg ha^−1^). P was applied as a basal fertilizer only at a rate of 75 kg P_2_O_5_ ha^−1^. K was equally split-applied as a basal and panicle fertilizer at a total rate of 105 kg K_2_O ha^−1^. Water was managed in the sequence of flooding, mid-season drainage, re-flooding, and moist intermittent irrigation. Weeds, pests, and diseases were intensively controlled by chemicals to avoid yield loss.

Rice plants were sampled at maturity with 10 hills from each high and moderate-density plot and 6 hills from each low-density plot. The plant samples were hand threshed. Filled spikelets were separated from unfilled spikelets by submerging them in tap water. Three subsamples of 30 g filled spikelets and 15 g unfilled spikelets were collected to count spikelet number. Dry weight of the filled spikelets was determined after oven-drying at 70 °C to constant weight. Spikelet filling percentage and grain weight were calculated. Grain yield was determined from a 5 m^2^ area in each plot and adjusted to a moisture content of 14%.

Data were analysed by analysis of variance (ANOVA) and the least significant difference (LSD) test at the 0.05 probability level (Statistix 8.0, Analytical Software, Tallahassee, FL, USA). The relationships between yield attributes, hill density, and N rate were evaluated by using multiple linear regression with the model of y = a + bx + cz, where y is the yield attribute, x is the hill density, z is the N rate, a is the constant coefficient, and b and c are the variable coefficients. The statistical significance was set at the 0.05 probability level.

## Data Availability

All data generated or analysed during this study are included in the article.

## References

[1] Hsiaoping, C. Rice consumption in China: Can China change rice consumption from quantity to quality in *Rice is life: scientific perspectives for the 21st century* (eds Toriyama, K., Heong, K. L. & Hardy, B.) 497–499 (International Rice Research Institute, 2005).

[2] Peng S, Tang Q, Zou Y (2009). Current status and challenges of rice production in China. Plant Prod. Sci..

[3] Yang W, Peng S, Laza RC, Visperas RM, Dionisio-Sese ML (2008). Yield gap analysis between dry and wet season rice crop grown under high-yielding management conditions. Agron. J..

[4] Jiang P (2016). Potential yield increase of hybrid rice at five locations in southern China. Rice.

[5] Liu Q, Wu X, Chen B, Ma J, Gao J (2014). Effects of low light on agronomic and physiological characteristics of rice including grain yield and quality. Rice Sci..

[6] Yang C, Xu Y (2014). Analysis of the August 2014 atmospheric circulation and weather. Meteorol Monthly.

[7] Tie X (2016). Effect of heavy haze and aerosol pollution on rice and wheat productions in China. Sci. Rep..

[8] Zhou L, Chen X, Tian X (2018). The impact of fine particulate matter (PM2.5) on China’s agricultural production from 2001 to 2010. J. Clean. Prod..

[9] Kobata T, Sugawara M, Takatu S (2000). Shading during the early grain filling period does not affect potential grain dry matter increase in rice. Agron. J..

[10] Wei H (2018). Combined effect of shading time and nitrogen level on grain filling and grain quality in *japonica* super rice. J. Integr. Agr..

[11] Huang M (2013). Tillering responses of rice to plant density and nitrogen rate in a subtropical environment of southern China. Field Crops Res..

[12] Huang M, Chen J, Cao F, Zou Y (2018). Increased hill density can compensate for yield loss from reduced nitrogen input in machine-transplanted double-cropped rice. Field Crops Res..

[13] Fan M (2012). Improving crop productivity and resource use efficiency to ensure food security and environmental quality in China. J. Exp. Bot..

[14] Liu X (2013). Enhanced nitrogen deposition over China. Nature.

[15] Guo JH (2010). Significant acidification in major Chinese croplands. Science.

[16] Le C (2010). Eutrophication of lake waters in China: Cost, causes, and control. Environ. Manage..

[17] Yin X, Huang M, Zou Y (2018). Changes in rice yield stability in southern China from 1949 to 2015. Agric. Environ. Lett..

[18] Thomas EV (2002). Development of a mechanism for transplanting rice seedlings. Mech. Mach. Theory.

[19] Yang J, Zhang J (2006). Grain filling of cereals under soil drying. New Phytol..

[20] Yang J, Zhang J (2010). Crop management techniques to enhance harvest index in rice. J. Exp. Bot..

[21] Nakano H, Morita S, Kitagawa H, Wada H, Takahashi M (2012). Grain yield response to planting density in forage rice with a large number of spikelets. Crop Sci..

[22] Kropff, M. J., Cassman, K. G., Peng, S., Matthews, R. B. & Setter, T. L. Quantitative understanding of yield potential in: *Breaking the yield barrier* (ed. Cassman, K. G.) 21–38 (International Rice Research Institute, 1994).

[23] Kamiji Y, Yosida H, Palta JA, Sakuratani T, Shiraiwa T (2011). N applications that increase plant N during panicle development are highly effective in increasing spikelet number in rice. Field Crop Res..

